# Projected Estimates of Opioid Mortality After Community-Level Interventions

**DOI:** 10.1001/jamanetworkopen.2020.37259

**Published:** 2021-02-15

**Authors:** Benjamin P. Linas, Alexandra Savinkina, R. W. M. A. Madushani, Jianing Wang, Golnaz Eftekhari Yazdi, Avik Chatterjee, Alexander Y. Walley, Jake R. Morgan, Rachel L. Epstein, Sabrina A. Assoumou, Sean M. Murphy, Bruce R. Schackman, Stavroula A. Chrysanthopoulou, Laura F. White, Joshua A. Barocas

**Affiliations:** 1 Section of Infectious Diseases, Boston Medical Center, Boston, Massachusetts; 2 Boston University School of Medicine, Boston, Massachusetts; 3 Clinical Addiction Research and Education Unit, Section of General Internal Medicine, Department of Medicine, Boston University School of Medicine, Boston, Massachusetts; 4 Grayken Center for Addiction at Boston Medical Center, Boston, Massachusetts; 5 Boston University School of Public Health, Boston, Massachusetts; 6 Department of Healthcare Quality and Research, Weill Cornell Medical College, New York, New York; 7 School of Public Health, Brown University, Providence, Rhode Island

## Abstract

**Importance:**

The United States is experiencing a crisis of opioid overdose. In response, the US Department of Health and Human Services has defined a goal to reduce overdose mortality by 40% by 2022.

**Objective:**

To identify specific combinations of 3 interventions (initiating more people to medications for opioid use disorder [MOUD], increasing 6-month retention with MOUD, and increasing naloxone distribution) associated with at least a 40% reduction in opioid overdose in simulated populations.

**Design, Setting, and Participants:**

This decision analytical model used a dynamic population-level state-transition model to project outcomes over a 2-year horizon. Each intervention scenario was compared with the counterfactual of no intervention in simulated urban and rural communities in Massachusetts. Simulation modeling was used to determine the associations of community-level interventions with opioid overdose rates. The 3 examined interventions were initiation of more people to MOUD, increasing individuals’ retention with MOUD, and increasing distribution of naloxone. Data were analyzed from July to November 2020.

**Main Outcomes and Measures:**

Reduction in overdose mortality, medication treatment capacity needs, and naloxone needs.

**Results:**

No single intervention was associated with a 40% reduction in overdose mortality in the simulated communities. Reaching this goal required use of MOUD and naloxone. Achieving a 40% reduction required that 10% to 15% of the estimated OUD population not already receiving MOUD initiate MOUD every month, with 45% to 60%% retention for at least 6 months, and increased naloxone distribution. In all feasible settings and scenarios, attaining a 40% reduction in overdose mortality required that in every month, at least 10% of the population with OUD who were not currently receiving treatment initiate an MOUD.

**Conclusions and Relevance:**

In this modeling study, only communities with increased capacity for treating with MOUD and increased MOUD retention experienced a 40% decrease in overdose mortality. These findings could provide a framework for developing community-level interventions to reduce opioid overdose death.

## Key Points

**Question:**

Using simulated urban and rural communities, what evidence-based practices are associated with a reduction in opioid overdose mortality of at least 40% by 2022?

**Findings:**

In this decision analytical model using simulated urban and rural communities, no single intervention or approach was associated with a 40% reduction in overdose mortality in any community. Achieving a 40% reduction required increasing capacity for treating with medications for opioid use disorder, improving retention on medications, and increased naloxone distribution.

**Meaning:**

These findings suggest that reducing opioid overdose may require substantial, coordinated effort with a focus on improving initiation with medications, retention in care, and increased naloxone distribution.

## Teaser

This decision analytical model examines the association of evidence-based practices alone or in combination with reduction of opioid overdose mortality by at least 40% in simulated communities.

## Introduction

The United States is facing a crisis of opioid-related overdose.^1^ At this time, more people die of overdose every year than died of AIDS at the peak of the HIV epidemic.^2^ Communities seek effective responses to prevent opioid death.

Medications for opioid use disorder (MOUD) reduce risk of opioid overdose mortality.^3,4,5,6^ However, the benefits of MOUDs diminish when individuals discontinue medications.^4,5,7^ Therefore, to improve overdose rates more people need to both start and continue use of MOUD. Community overdose prevention education and the naloxone rescue kit distribution is another response that is associated with reduced overdose death.^8,9,10,11,12^

Other approaches to treating opioid use disorder (OUD), including acute drug detoxification and abstinence-based residential drug treatment, have not been shown to be effective and can be harmful.^6,13^ Therefore, such abstinence-based programs are not components of evidence-based intervention.

The National Institutes of Health and Substance Abuse and Mental Health Services Administration HEALing Communities Study has set a goal of reducing opioid overdose deaths in US communities by at least 40% by 2022.^14^ Limited evidence exists to guide communities as they set priorities for implementing local responses. Understanding the combination of evidence-based approaches needed to achieve a 40% reduction is vital. Therefore, we used simulation modeling to investigate combinations of community-level interventions to determine their association with overdose rates.

## Methods

This study was reviewed by the Boston University Medical Campus Institutional Review Board and was deemed not to be human participants research and therefore exempt from approval and informed consent. This study followed the and Consolidated Health Economic Evaluation Reporting Standards (CHEERS) reporting guideline.

### Overview of the RESPOND Model

#### Overview

We used the Researching Effective Strategies to Prevent Opioid Death (RESPOND) model, a dynamic population state-transition model of OUD and OUD treatment, to simulate the population with OUD living in Massachusetts from 2015 to 2022. We estimated the combined outcomes associated with evidence-based, multicomponent community-level interventions to prevent overdose.

To improve the generalizability and usefulness of the results, we completed the analysis from the perspective of 8 Massachusetts community archetypes, intended to reflect OUD epidemiological characteristics, treatment outcomes, and naloxone use in a variety of urban and rural communities. We characterized these archetypes along 2 axes: (1) current initiation rates of outpatient MOUD (ie, buprenorphine, methadone, naltrexone), and (2) current distribution of naloxone. We defined 4 urban and 4 rural archetypes and generated results for all 8 community types (Table 1).

**Table 1.** Definitions of Community Archetypes Used for a Simulation of Community-Level Response to the Opioid Overdose Epidemic

Community archetype	Opioid population engaged with MOUD, %	6-mo retention on buprenorphine-naloxone, %
**Urban**		
Status quo 6-mo retention on buprenorphine = 33%; ratio of naloxone distributed: kits used per 1 attempted rescue = 77 (distribution to PWUD), 247 (community distribution)		
Low MOUD/low naloxone	10	33
Low MOUD/high naloxone	10	33
High MOUD/low naloxone	15	33
High MOUD/high naloxone	15	33
**Rural**		
Status quo 6-mo retention on buprenorphine = 17%; ratio of naloxone distributed: kits used per 1 attempted rescue = 253 (distribution to PWUD), 594 (community distribution)		
Low MOUD/low naloxone	5	17
Low MOUD/high naloxone	5	17
High MOUD/low naloxone	10	17
High MOUD/high naloxone	10	17

Abbreviations: MOUD, medications for opioid use disorder; PWUD, persons who use drugs.

For each community, we initiated a simulation in calendar year 2015 and simulated that community’s population with opioid misuse or OUD in 2015 to 2020. In simulated calendar year 2020, we adjusted parameters to estimate the outcomes associated with implementing 3 strategies to prevent opioid misuse and overdose: (1) initiate more people to MOUD, (2) improve 6-month retention with MOUD, and (3) increase distribution of naloxone to the community. We then ran each simulation for 2 years (to calendar 2022) to project outcomes.

### Model Structure

**Figure 1.**
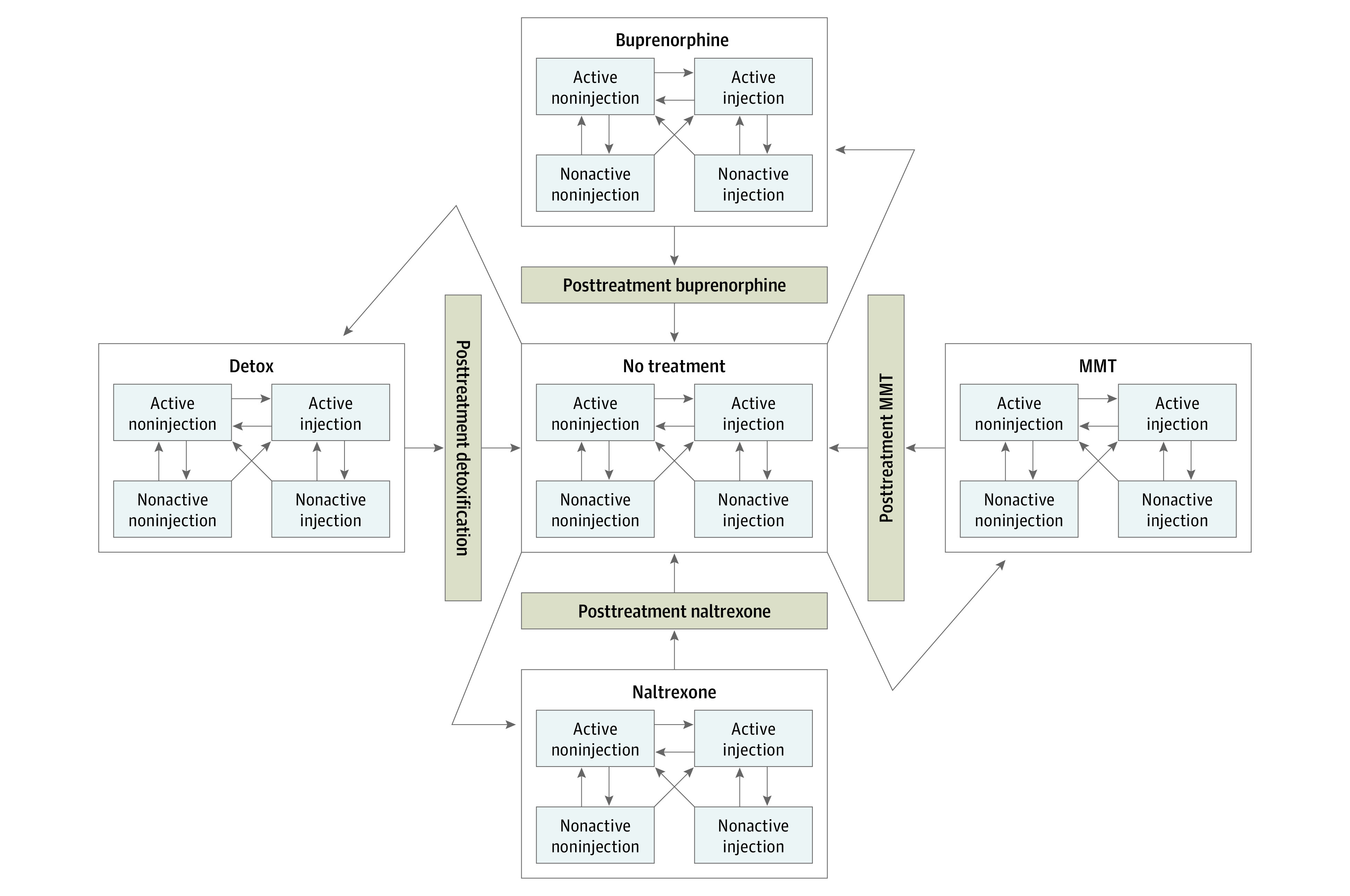
Model Structure of the Researching Effective Strategies to Prevent Opioid Death (RESPOND) Model MMT indicates methadone maintenance therapy.

The RESPOND model was developed in 2018 to 2020 (Figure 1). The model includes simulation of epidemiological characteristics, opioid use, overdose, and outpatient MOUD treatment with each of 3 Food and Drug Administration–approved medications: methadone, buprenorphine-naloxone, and injectable naltrexone.

#### Epidemiology

The simulation uses a weekly time step. In each week, the new population is added to the simulation, with independent rates of entry stratified by age and sex. The rates of addition represent the development of new OUD and emigration to the state; they are inputs to the model and are calibrated such that the simulated population matches the age and sex distribution of OUD in Massachusetts. When projecting future outcomes, the simulation assumes that epidemiological trends observed in the years leading up to the start of the simulation continue throughout the rest of the simulation.

#### Opioid Use

The simulation categorizes persons with OUD into 4 states representing all permutations of current vs no current opioid use, and noninjection vs injection opioid use (Figure 1). Only the population currently using opioids is eligible to initiate MOUD and is exposed to overdose risk. Injection drug use is characterized by a higher incidence of opioid overdose than noninjection use, as well as higher risk of death from nonoverdose causes (eg, infections associated with drug use). Movement occurs among all health states throughout the simulation, reflecting the relapsing and remitting nature of OUD (Table 2).

**Table 2.** Select Input Parameter Values for a Simulation of Community-Level Response to the Opioid Overdose Epidemic

Parameter	Value	Range evaluated	Source
Population demographic and epidemiological characteristics			
Men, %	58.4	NA	MDPH,^15^ 2017
Population using MOUD at baseline, %	15.5	5-15.5	MDPH,^15^ 2017
Population with injection drug use at baseline, %	25	NA	CBHSQ,^16^ 2016
Population actively using opioids at baseline, %	83.8	NA	CBHSQ,^16^ 2016
Annual new OUD, mean, No.^a^	8164-44 564		MDPH,^15^ 2017
Monthly MOUD treatment starts, No. per 10 000 OUD population^a^			
Buprenorphine	252	169-2519	MDPH,^15^ 2017
Methadone	74	49-736	MDPH,^15^ 2017
Naltrexone	29	19-287	MDPH,^15^ 2017
6-mo retention on MOUD treatment, mean, %^b^			
Buprenorphine	34	17-81	Morgan et al,^7^ 2018
Methadone	56	28-89	Soyka et al,^17^ 2008
Naltrexone	21	11-73	Morgan et al,^7^ 2018
Fatal overdoses, proportion of total overdoses	0.1373	0.1099-0.1609	MDPH,^15^ 2017

Abbreviations: CBHSQ, Center for Behavioral Health Statistics and Quality; MDPH, Massachusetts Department of Public Health; MOUD, medications for opioid use disorder; OUD, opioid use disorder.

^a^ Archetype communities were defined by percentage of OUD population using MOUD at baseline, monthly MOUD treatment starts, mean MOUD retention at 6 months. Listed ranges evaluated include lowest to highest parameter variables used in archetype communities.

^b^ Varies annually.

#### Overdose

Among the population with current opioid use, there is a risk in every time step of opioid overdose. The risk is stratified by route of administration (injection vs noninjection) and also by age and sex. Among those who experience opioid overdose, there is a risk of death from that overdose (Table 2).

#### Outpatient MOUD Treatment

Among the population that is currently using opioids, there is a probability in every time step of transitioning to outpatient MOUD treatment with either methadone, buprenorphine-naloxone, or injectable naltrexone (Figure 1). Each treatment has the same simulation structure. Among the population that initiates MOUD, a proportion immediately transitions to no current drug use. Subsequently, bidirectional movement occurs between current and no current drug use states. MOUD initiation affects drug use transition rates such that the net effect decreases the likelihood of transitioning to or staying in a current drug use state. MOUD also has an independent effect on overdose, such that among the population that is currently using opioids while continuing to use MOUD, there is a decreased risk of opioid overdose compared with the risk among those who are actively using opioids and not using MOUD.

### Data and Parameter Estimation

The primary data source for RESPOND is the Massachusetts Public Health Data Repository,^18^ a longitudinally linked administrative records database that includes service encounter data from more than 16 agencies in the Commonwealth of Massachusetts. The database includes approximately 97% of the individuals in Massachusetts, and data across agencies is linkable at the person-level. We used data from the Massachusetts Public Health Data Repository to estimate the demographic characteristics associated with OUD in Massachusetts, rates of treatment seeking, and overdose. We used published characterizations of the natural history of OUD to estimate transitions between OUD states without treatment. We used clinical trials data to estimate treatment parameters (Table 2).

#### Epidemiological Characteristics

We used the Massachusetts Public Health Data Repository to estimate the total size of the OUD population in Massachusetts from 2015 to 2020, the size of the arriving population with OUD, and the age and sex of individuals with OUD in Massachusetts (Table 2). To estimate population size, including the population with occult or unidentified opioid use, we used a capture-recapture method, which we have previously published.^19^ Capture-recapture is a method for indirect population measurement. The method uses linked data sources to estimate the distribution of how often individuals are captured (ie, included) in the various linked databases. Some individuals appear in only 1 database, while others appear in 2 or more. Using probability theory, it is possible to interpolate the likely number of individuals who exist but were not captured by any database.

#### Opioid Use and Overdose

We used the scientific literature to estimate transitions between opioid use states without treatment (Table 2).^20,21,22^ To estimate the rate of opioid overdose among those with OUD, we used age- and sex-stratified Massachusetts Public Health Data Repository overdose counts in the numerator, and age and sex strata population estimates in the denominator. We also used the Massachusetts Public Health Data Repository to estimate the proportion of all opioid overdoses that are fatal (Table 2).

#### Outpatient MOUD Treatment

We used National Institute on Drug Abuse Clinical Trials Network data to estimate transitions between opioid use states while using MOUD.^23,24^ We analyzed urinalysis data from the Clinical Trials Network in an as-treated manner, such that we could estimate both rates of relapse and remission while continuing to use MOUD. To estimate transition parameters, we employed a multi-state Markov model. We provide details in the eAppendix in the Supplement.

#### Naloxone

Naloxone affects the simulated probability of death conditional on having experienced an opioid overdose. We estimate the impact of the number of naloxone kits distributed on the simulated conditional probability of overdose using the approach of Irvine et al^25^:

*Fatal overdoses prevented* = *N_kit_*_s_ × *kits distributed per rescue attempt* × *attempts per fatal overdose prevented*

With this estimate of fatalities prevented by a given number of kits distributed, we adjusted downward the number of fatal overdoses expected. The conditional probability of death given opioid overdose in the presence of higher naloxone distribution is then:


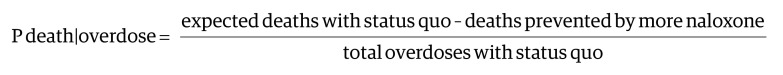


### Simulation Scenarios and Approaches to Characterizing Community Archetypes

#### Urban vs Rural Communities

We identified Massachusetts counties as being predominantly rural or urban based on census data.^26^ We characterized urban and rural simulated communities in 2 ways based on data from the scientific literature and Massachusetts Public Health Data Repository. First, urban communities have higher retention in MOUD treatment assuming the status quo (Table 1).^27,28,29,30^ Second, urban communities require fewer naloxone kits be distributed per attempted rescue (Table 1).

#### MOUD Use at Baseline

For both urban and rural settings, we simulated low and moderate levels of current MOUD use at baseline. For urban communities, we set low MOUD use to be similar to the proportion of people with OUD who are actively prescribed MOUD in national medical claims data.^7^ We set moderate MOUD use in urban areas at baseline to be equal to that observed in the state-wide Massachusetts Public Health Data Repository.

To simulate low and moderate MOUD use in rural areas, we used the Massachusetts Public Health Data Repository to estimate the proportion of the population engaged with MOUD in rural Massachusetts counties.

#### Naloxone Use at Baseline

We used data informing the number of naloxone kits distributed by the Massachusetts Department of Public Health to each county in Massachusetts, as well as county-specific report-back rates of rescue attempts when individuals picked-up new naloxone kits. We simulate low naloxone use based on naloxone distribution in 2013 (the beginning of the simulation timeframe). Simulation of high naloxone use is based on 2018 distribution numbers.

### Data Analysis

We simulated the population with OUD in each community archetype to project overdoses and treatment capacity needs at 2 years, assuming the status quo, each of the 3 interventions alone, and combinations of all 3 interventions at various levels of intensity and effectiveness. For each scenario, we projected overdose fatalities, treatment capacity, and naloxone distribution requirements after 2 years. We report results in terms of relative reduction in overdose fatalities compared with what would have been expected in a counter-factual of no intervention. We do not report absolute event counts, because the archetype communities are hypothetical. Data were analyzed from July to November 2020.

## Results

**Figure 2.**
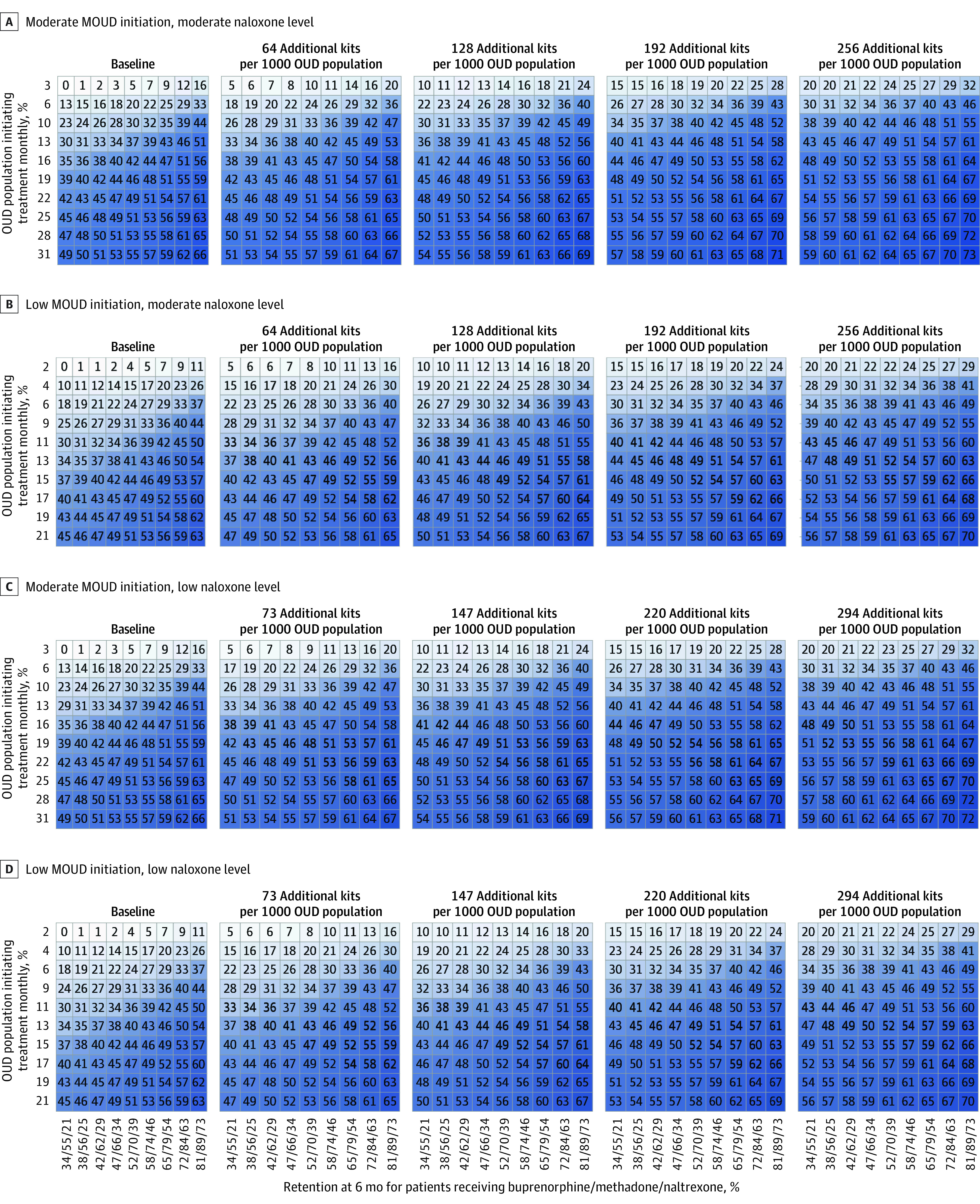
Three-Way Sensitivity Analyses of Community-Level Interventions to Improve Initiation and Retention With Medications for Opioid Use Disorder (MOUD) and Increase Naloxone Distribution in Urban Communities Each panel represents an urban community archetype, defined in terms of baseline rates of initiation with MOUD and use of naloxone. Within a panel, the vertical axis represents the rate of initiation with MOUD achieved by an intervention, expressed in terms of the percentage of the total opioid use disorder (OUD) population that is initiating MOUD each month. The horizontal axis shows the absolute 6-month retention proportion for each MOUD (ie, buprenorphine, methadone, or naltrexone). To read a panel, choose a rate of initiation and draw a line horizontally from the vertical axis to the right. Choose a level of retention and draw a vertical line upward. Where those lines intersect, the number in the cell and the color shade represent the percentage decrease in overdose mortality at 2 years compared with the counterfactual of no intervention. Moving across the page shows the results with increasing rates of naloxone distribution.

**Figure 3.**
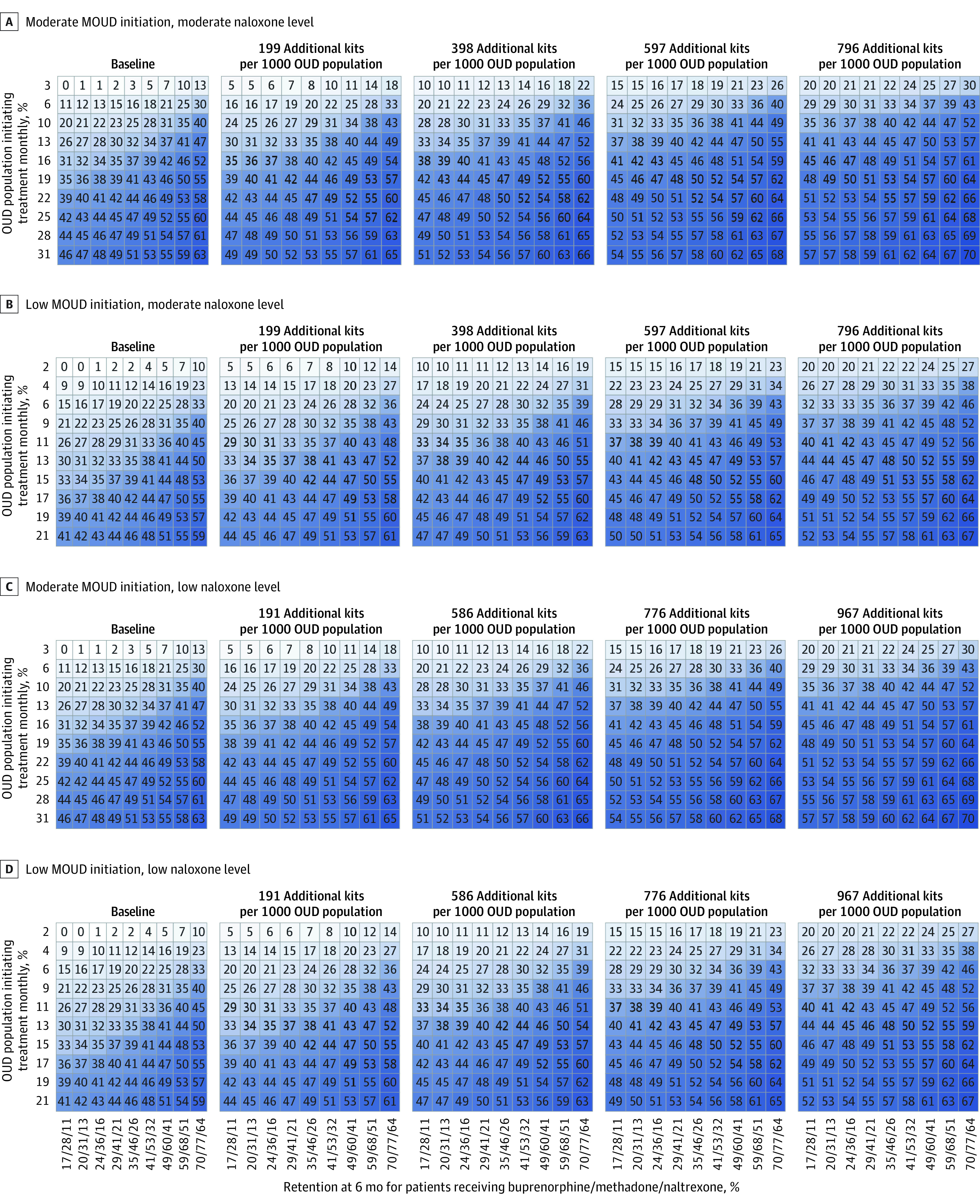
Three-Way Sensitivity Analyses of Community-Level Interventions to Improve Initiation and Retention With Medications for Opioid Use Disorder (MOUD) and Increase Naloxone Distribution in Rural Communities Each panel represents a rural community archetype, defined in terms of baseline rates of initiation with MOUD and use of naloxone. Within a panel, the vertical axis represents the rate of initiation with MOUD achieved by an intervention, expressed in terms of the percentage of the total opioid use disorder (OUD) population that is initiating MOUD each month. The horizontal axis shows the absolute 6-month retention proportion for each MOUD (ie, buprenorphine, methadone, or naltrexone). To read a panel, choose a rate of initiation and draw a line horizontally from the vertical axis to the right. Choose a level of retention and draw a vertical line upward. Where those lines intersect, the number in the cell and the color shade represent the percentage decrease in overdose mortality at 2 years compared with the counterfactual of no intervention. Moving across the page shows the results with increasing rates of naloxone distribution.

Overall, simulated community-level interventions reduced overdose mortality, but no single intervention was associated with a 40% reduction in overdose death. Figure 2 and Figure 3 provide an overview of the results in the simulated urban and rural communities.

### Urban Communities

Increasing MOUD initiation and retention in care and expanding naloxone distribution all was associated with a decreased rate of overdose in simulated urban communities, but no single intervention was associated with achievement of the target of a 40% reduction in overdose within 2 years, even when assuming large improvements from the status quo (Figure 2). For example, consider an urban community with few people using MOUD and little naloxone distribution (Figure 2B). Were such a community to focus solely on MOUD initiation, it would need to increase MOUD initiation by 8-fold, such that in every month, 17% of the eligible population not on treatment was seeking care.

However, there were substantial synergies with multiple interventions. For example, consider the same urban community with little treatment or naloxone at baseline. If it was possible to improve 6-month retention with buprenorphine to 65%, methadone to 79%, and naltrexone to 54%, then it would be possible to reduce overdose fatality by 40% if in every month 11% (instead of 17%) of the eligible population not currently receiving treatment initiated an MOUD. If efforts to improve initiation and retention with MOUD were coupled with distribution of an additional 220 naloxone kits per 1000 persons with OUD, then that community would need to increase MOUD initiation such that only 6% of the eligible population initiated an MOUD each month.

The specific estimated reductions in overdose death differed for each community archetype, but combination interventions were essential in every community to make a 40% reduction in overdose feasible. In general, all urban community types required at least 10% of eligible people initiating MOUDs in every month and 50% retention with MOUDs at 6 months. In all urban community types, naloxone played a substantial role in reducing overdose mortality, but there was no feasible strategy that was associated with reduction in opioid overdose by 40% using only naloxone without including MOUD.

### Rural Communities

In simulated rural communities, achieving a 40% reduction in overdose required higher rates of treatment initiation, treatment retention, and naloxone distribution than in urban areas. This finding reflects lower rates of retention in care and the requirement for more naloxone to be distributed per reported rescue attempt in rural areas. Additionally, because treatment initiation rates are generally lower in rural than in urban areas, there was less of a synergistic effect between treatment initiation and retention.

Similar to urban communities, no single intervention type was associated with a 40% reduction in overdose mortality in rural communities. Improving both initiation of and retention with MOUD provided some synergies, and it was only possible to achieve a 40% reduction in overdose rates in rural communities through a combined effort that used MOUD and naloxone. In general, all rural community archetypes required at least 10% of eligible people initiating MOUD in every month and 50% retention on MOUD at 6 months.

## Discussion

This decision analytical model used the RESPOND model to project the outcomes associated with community-level interventions to prevent opioid overdose in simulated rural and urban communities. These results are intended to be useful to community public health and policy decision-makers who are allocating resources to respond to the opioid overdose crisis. Our findings bring several important messages to light. First, there was no feasible single intervention associated with a 40% reduction in overdose in any community. Preventing overdose deaths required treating people with OUD with medications, such that they use opioids less often and are at lower risk of overdose death, in addition to overdose education and naloxone distribution. In some settings, opioid agonist medications (ie, methadone and buprenorphine) are controversial owing to stigma.^31^ In such settings, expanding naloxone distribution can be an appealing response because it can save lives without requiring decisions about treatment capacity that address stigma associated with MOUD. However, our results demonstrate that focusing solely on naloxone rescue may not achieve overdose prevention goals.

Another important finding for urban and rural areas is the critical role of retention in care in achieving overdose reduction results. Explicit discussion of improving retention as a public health measure is beginning to emerge.^32^ Contingency management,^33^ eliminating abstinence and counseling requirements,^34^ removing time-limits on insurance coverage,^35^ and treating concomitant mental health problems^36,37^ are all strategies to improve MOUD retention in care.^38^ Long-acting depot formulation injections may also improve retention, but evidence to date is limited.^7^ Although improving retention is challenging, ending the overdose crisis requires improving retention in care through better pharmaceutical agents, peer navigation, and other support services.

The synergies among interventions were less marked in our simulated rural settings than in simulated urban areas, likely because many rural areas had lower baseline MOUD initiation and retention rates and less naloxone distribution than urban areas, which means that there are fewer synergies to leverage. The implication is that rural areas may prioritize increasing MOUD initiation rates before working to improve retention, effectively priming the pump for retention efforts. However, the risk of such a 2-staged intervention approach is that retention efforts do not follow the initial work to improve treatment initiation, losing a great deal of potential benefit.

Finally, these results define new milestones by which communities can measure progress toward reducing overdose deaths. Although the relative increase in MOUD initiation and retention needed in each community differed based on the baseline level of MOUD use, in all communities achieving a 40% reduction in opioid overdose deaths required that a minimum of 10% of the total eligible population not in treatment start treatment every month and that approximately 50% was retained at 6 months. These certainly are not rigid measures, but they provide some “road signs” that communities can use to stay on track.

### Limitations

There are limitations to this work. First, the quantitative results are intended to provide qualitative insights. The goal of the analysis is not to forecast precise overdose rates, nor define rigid implementation targets. It would be a mistake for a community to identify itself among the archetypes, choose a strategy and targets, and assume that this analysis provides the exact recipe for success.

Second, the analysis attempts to be generalizable while exploring specific contexts. It is not possible to generate forecasts for every community in the US, and there is no common definition of what makes a community. At the same time, it is not possible to inform the US opioid epidemic response without recognizing the importance of community context. The archetype results that we report strive to achieve the appropriate balance. Decision-makers in contexts outside of Massachusetts could use these data to understand the relative potential contributions associated with increasing MOUD initiation and retention and naloxone distribution in their communities. While projections of total counts of overdoses and treatment start at various time points clearly depend on local population size and OUD prevalence, the high-level messages we highlight are likely robust and are actionable data.

Third, we do not include overdose prevention sites (eg, supervised consumption sites) along with MOUD and naloxone as community-level interventions. A modeling study focused on British Columbia found that such sites were important.^25^ Because overdose prevention and supervised drug consumption sites are not currently legal in the US, we did not include them as overdose-averting interventions.

Fourth, the simulation does not include race/ethnicity, because we are not able to stratify key simulation inputs by age, sex, and race/ethnicity simultaneously. Overdose mortality differs by race/ethnicity, as have changes in overdose rates. Because MOUD options are often segregated by race and ethnicity,^39^ a strategy focused solely on numerical goals without a concomitant focus on equity could leave specific communities behind.

## Conclusions

These findings suggest that reducing opioid overdose may require simultaneous scale-up of MOUD, improvement in retention with MOUD, and increased naloxone distribution. As a starting point, communities around the country likely need to be prepared to have at least 10% of their estimated OUD population initiating MOUD every month and 50% of those persons remaining in care for at least 6 months. While that task is not trivial, it is unlikely that a community could achieve significant reductions in opioid overdose without significant investment in a multistrategy approach.

## Notes

Supplement. **eAppendix.** Supplementary Methods **eReferences.**
