# Supplementary material for: Projected Estimates of Opioid Mortality After Community-Level Interventions
Source: JAMA Netw Open. 2021 Feb 15;4(2):e2037259. doi: 10.1001/jamanetworkopen.2020.37259 (PMC7885041; doi:10.1001/jamanetworkopen.2020.37259)
Supplement: Supplement. — eAppendix. Supplementary Methods eReferences. [file jamanetwopen-e2037259-s001.pdf]

Supplemental Online Content

Linas BP, Savinkina A, Madushani RWMA, et al. Projected estimates of opioid mortality after community-level interventions. *JAMA Netw Open*. 2021;4(2):e2037259.
doi:10.1001/jamanetworkopen.2020.37259

eAppendix. Supplementary Methods
eReferences

This supplemental material has been provided by the authors to give readers additional information about their work.

© 2021 Linas BP et al. *JAMA Network Open.*

eAppendix. Supplementary Methods

TABLE OF CONTENTS

INTRODUCTION ... 3

MODEL STRUCTURE ... 4

Overview ... 4

Key Data Sources for the Model ... 6

Massachusetts Public Health Data Repository ... 6

NIDA Clinical Trial Network Protocols 0051 (CTN) ... 6

Medical Literature ... 6

Components ... 6

Population Dynamics ... 6

Initial Cohort ... 6

Aging ... 8

Entering Cohort ... 8

Natural History of OUD ... 10

Care Delivery ... 12

Movement From no Treatment to Treatment Episodes ... 12

Block Initiation Effect ... 14

Transitions Between Active and Non-Active Opioid Use While Engaged With Treatment ... 15

Probability of Loss to Follow-Up ... 17

Overdose ... 19

No Treatment ... 20

Overdose While on Treatment ... 21

Overdose During the Post-Treatment Period ... 22

Mortality ... 23

Fatal Overdose ... **Error! Bookmark not defined.**

Non-Overdose Mortality ... **Error! Bookmark not defined.**

REFERENCES ... 25

INTRODUCTION

The growing prevalence of Opioid Use Disorder (OUD) has resulted in an increase in opioid overdoses in the United States. Overdose is the leading cause of premature death among Americans under the age of 50 and has increased by more than 2.5 times between 1999 and 2015. Although evidence-based treatments are available for treating OUD, these treatments are under-utilized, thus the impact of opioids on the United States' population persists.

Researchers and policy makers have made efforts to create feasible action plans for reducing the prevalence of OUD. Unfortunately, most policy makers do not have the evidence needed for informing and implementing system-level change. System-level thinking investigates how systems operate and how they can be modified to produce desired outcomes. At this time, data on system-level interventions for OUD are limited and inconsistent.

In an effort to fill the knowledge gap, simulation modeling can be used to integrate data from multiple sources to translate outcomes from clinical studies to policy-relevant data about population health and cost. By simulating state-level behaviors and practices related to OUD, we can project and evaluate the impact of relevant interventions and policies on public health outcomes and costs, hence informing practice and policy decisions to combat OUD.

The **R**esearching **E**ffective **S**trategies to **P**revent **O**pioid **D**eath (RESPOND) model is a state-transition, cohort-based model that simulates populations with high-risk opioid use in a state, including the natural history of opioid use disorder, movement on and off of opioid treatment, and overdose. The model provides outputs and projections that decision-makers can use to evaluate and modify care delivery systems to match their local epidemics and available resources.

Model inputs and parameters are adaptable to users' needs, namely to represent heterogeneous populations, different dynamics of the drug overdose epidemic, and the effectiveness of intervention strategies in the prevention of opioid-related harms. The user, for example, can customize among other things, the demographics, time in each cycle, transition probabilities between health statuses and treatment states, and the number of health states included in the model in order to represent different structure and disease dynamics of the underlying population.

© 2021 Linas BP et al. *JAMA Network Open.*

MODEL STRUCTURE

Overview

RESPOND is a state-transition, cohort-based [1, 2] model that simulates the population living within a jurisdiction and who have high-risk opioid use. Typically, RESPOND simulates the population of a state, but it can also simulate a smaller area, such as a town or rural community, depending on the model parameter values. The model employs a Markov process with a weekly cycle length to accurately reflect population dynamics, clinical progression, and treatment of opioid use disorder.

The model structure comprises four main components; 1) population dynamics, 2) natural history of OUD, 3) care delivery, and 4) mortality.

The first part of the model pertains to simulating the epidemiology and demography of the opioid epidemic assuming either an open or closed cohort, namely allowing or not allowing new individuals to enter ("arrivals") the simulation process at each cycle respectively. When simulating an open cohort, the arrival rate represents both the development of new opioid use disorder, and migration into the state among those with existing opioid use. RESPOND does not directly simulate the development of opioid use disorder. Instead, the population "arrives" in each time step as an exogenous rate without simulating the specific steps in the development of opioid use. The input values for arrivals are calibrated to match observed, state-level OUD estimates stratified by age and sex.

The second part (core simulation - Figure 1) of the RESPOND model involves the simulation of the natural history of opioid use disorder as a relapsing and remitting disease over a lifetime. RESPOND simulates OUD as a series of transitions between four health states of opioid use: 1) active, non-injection, 2) non-active, non-injection, 3) active injection, and 4) non-active, injection opioid use. In each time-step of the simulation, population fractions move between opioid use states. The definitions of "active" and "injection" opioid use can vary (but must be pre-specified) depending on the users' needs and available information. In the RESPOND Massachusetts base case, "active" opioid use is defined as any reported use in the previous seven days. "Injection" opioid use reflects *any* injection in the preceding seven days (a person who is both injecting and using oral opioids would be categorized as "injection" in RESPOND).

The third part of RESPOND is related to care delivery, and currently includes four "treatment episodes": 1) outpatient buprenorphine (Bup), 2) outpatient injectable naltrexone (Ntx), 3) outpatient methadone (Mmt) maintenance, and 4) inpatient acute drug detoxification center. The model is adaptable to additional intervention types to better reflect local conditions and evolutions in the treatment field. In

© 2021 Linas BP et al. *JAMA Network Open.*

general, treatment episodes tend to decrease movement into active drug use, increase movement into non-active drug use, and have an independent effect on overdose rates conditional on active drug use.

The fourth part of the model is mortality. RESPOND simulates overdose-related mortality, and competing causes of death. RESPOND simulates overdose mortality by first simulating overdose incidence as a function of age and type of drug use (injection vs. non-injection use). Next, the model simulates a probability of death conditional on having had an opioid overdose. The model simulates competing causes of death through the use of standardized mortality ratios that are a function of age, sex, and type of opioid use (injection vs. non-injection).

The primary model outputs are: 1) All-cause mortality, 2) Overdose mortality, and 3) Number of people on treatment.

The simulation process is as follows: At simulation start, the model initiates a cohort of people currently living with OUD in the jurisdiction of interest. Based on data from that jurisdiction, the model assigns the current population to a drug use state, as well as a treatment block, such that the simulated population, including the prevalence of OUD treatment, reflects the status quo. Moving forward through simulated time, the sequence of simulation steps are: 1) aging of the population, 2) arrival of new population, 3) transition between OUD drug use states, 4) transitions into and out of treatment, 5) overdose, and 6) death. At the end of this sequence of processes, the model advances simulated time by one cycle (week) and repeats the process. The simulation continues until a time horizon assigned by the user.

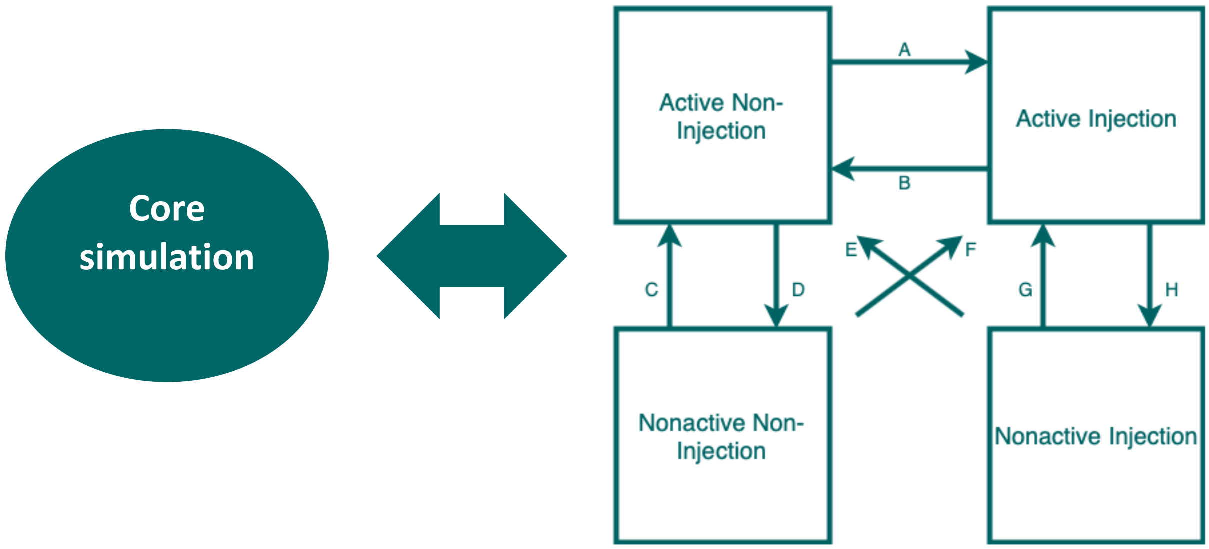

Figure 1. Core Simulation

© 2021 Linas BP et al. *JAMA Network Open.*

Key Data Sources for the Model

Massachusetts Public Health Data Repository

The Massachusetts Public Health Data Repository (MA PHD) is a linked longitudinal records dataset that includes administrative records and billing claims from over 15 state agencies. The spine of the database is the Massachusetts All Payers Claims database, which includes medical billing for all payers in the state. Because Massachusetts has near universal healthcare coverage, the MA PHD contains >97% of the residents of the state. The database links person-level records from vital statistics, the Department of Corrections, Emergency Medical Services, and the Bureau of Substance Addiction Services, such that it is possible to construct longitudinal, person-level trajectories across various treatment episodes, admissions to the hospital, and overdose events [3]. RESPOND uses the MA PHD dataset to estimate parameters such as OUD epidemiology in MA and rates of transition onto treatments assuming the status quo.

NIDA Clinical Trial Network Protocols 0051 (CTN)

The National Institute on Drug Abuse administers a large clinical trials network for evaluation of treatments for substance use disorders. The Clinical Trial Network (CTN) 0051 protocol was a head to head comparative effectiveness trial of sub-lingual buprenorphine and injectable naltrexone for individuals with opioid use disorder who were accessing acute opioid detoxification services. RESPOND uses urine toxicology data from the trial to estimate transitions between substance use states while taking buprenorphine or naltrexone, as well as health care utilization among patients with OUD [1]. [4-6]

Medical Literature

In addition to the primary data sources listed above, RESPOND estimates many model parameters from the medical literature. The detailed explanation of model parameters below provides references to the relevant publications.

Components

Population Dynamics

Initial Cohort

To simulate the demography and OUD epidemiology in the underlying population, RESPOND requires the initial cohort to be specified as follows: 1) age and sex distributions of people with opioid use disorder, 2)

© 2021 Linas BP et al. *JAMA Network Open.*

proportion of people beginning in each drug-use state, and 3) proportion of people within each treatment episode.

Structural Assumptions:

- No population begins the simulation in a post-treatment block.
- RESPOND does not characterize the population by race or ethnicity.

Methodological Notes:

Table 1 presents the key parameters related to cohort initialization.

To estimate the prevalence of both identified and unidentified OUD in Massachusetts, we employed a capture-recapture methodology to the Massachusetts Public Health Data Repository (MA PHD) to estimate the prevalence of OUD in Massachusetts during calendar years 2012 to 2015 stratified by age and sex [7]. The capture-recapture approach provides a method to estimate the total population with high-risk opioid use, including those who have not been identified as being an opioid user and do not appear in medical claims or prevalence surveys. To make population estimates beyond 2015, we combined data sources to extend the MA PHD. First, we used the National Survey on Drug Use and Health (NSDUH) to estimate the longitudinal trend in OUD in the United States 2015-2018. Next, we applied the trend line from NSDUH to the MA PHD OUD prevalence measurements. This approach assumes that the trend in prevalence in MA is similar to the trend nationally, but it provides an estimate of total population count that is informed by MA data.

Table 1. Initializing Cohort Parameters

**Parameter**	**Value**	**Method**	**Years**	**Stratification**	**Time Varying**	**Source**
Population size, n					Yes	MA PHD analysis update to Barocas et al. [7]
Population of high-risk opioid use						
Total ($\hat{\mathbf{N}}_{\mathbf{OUD,t}}$)		Capture recapture	2012-2015	Age (3 groups*) & Sex	Yes	MA PHD analysis update to Barocas et al. [7]
By age-group		Observed	2012-2015	Age (18 groups**) & Sex	Yes	US Census 2010
Proportion with injection drug use	25.09%	Observed	2013	Age (3 groups*) & Sex	No	NSDUH
Proportion non-actively using	9%	Estimated			No	(various studies: 7-10.7%)
Abbreviations:						

© 2021 Linas BP et al. *JAMA Network Open.*

- MA DPH: Massachusetts Department of Public Health data
- NSDUH: National Survey on Drug Use and Health

* 3 age groups: 10 – 24, 25 – 44, 45 – 99
** 18 age groups: 5-year age-groups from 10-99

Aging

RESPOND simulates discrete time steps (rather than continuous time) and categorical age groups or "brackets" over the lifetime. The user can define the bounds of age groups so as to match the structure of the underlying population. Aging occurs as the population progresses to the next age group after a number of cycles that is determined by the size of the age brackets. The model employs a half-cycle correction and aging occurs in discrete steps, namely only at multiples of the age group size.

Structural Assumptions:

- The entire population of the last age bracket (95 to 100-year-olds) is removed from the simulation at each aging cycle and replaced by the population from the previous age bracket.

Entering Cohort

RESPOND can simulate either an open cohort (meaning that new individuals arrive to the population over the course of the simulation), or a closed cohort. When running the model to simulate an open cohort, arrivals to the population occur at every time step. Arrival rates are stratified by age and sex and vary over time to realistically reflect the incidence of new opioid use disorder and/or movement into the jurisdiction.

Structural Assumptions:

- All new populations enter the first block ("No Treatment" episode) and the first OUD state (currently active, non-injection).
- All new arrivals enter the simulation as active non-injectors under the "No Treatment" block but can transition to other feasible OUD states in the OUD transition module.

Methodological Notes:

Error! Reference source not found. and **Table 2** present the key parameters related to cohort initialization.

- The size of the entering cohort ($N_{enter,t}$) at each cycle (week) is defined as:

$$N_{enter,t} = \widehat{N}_{OUD,t} - (\widehat{N}_{OUD,t-1} - \widehat{N}_{FOD,t-1} - \widehat{N}_{D\_other,t-1}) \qquad (1)$$

© 2021 Linas BP et al. *JAMA Network Open.*

where $\widehat{N}_{OUD,t}$ is the estimated OUD population in year t, $\widehat{N}_{FOD,t-1}$ is the estimated number of fatal overdoses, and $\widehat{N}_{D\_other,t}$ is the estimated total number of deaths from other causes, in the previous year (t-1).

Table 1. Entering Cohort Parameters

Parameter	Description	Value/Distribution	Method	Stratification	Source
$N_{initial,t}$	Initial cohort size	215,918	Estimated from NB regression with age, sex, & 3 years as covariates	Time (t=2013, 14, 15)	MA PHD Repository Barocas et al.
$N_{enter,t}$	Entering cohort size	$\widehat{N}_{OUD,t} - \widehat{N}_{initial,2013}$	Calculated	Time (t=2013)	
$N_{enter,t}$		$\widehat{N}_{OUD,t} - (\widehat{N}_{OUD,t-1} - \widehat{N}_{FOD,t-1} - \widehat{N}_{D\_other,t-1})$	Calculated	Time (t=2014, 15)	
$\widehat{N}_{FOD,t}$	Fatal overdoses	$\sim$ Poisson($\lambda_{FOD,t}$)	Estimated		
$\widehat{N}_{D\_other,t}$	Non-FOD deaths	$\sim$ Poisson($\lambda_{D\_other,t}$)	Estimated		MA PHD
p_{jkt}	Entering cohort proportions	$\sim$ beta (a_{jkt}, b_{jkt})	- Observed Imputation of missing strata %	Age (j=1,..,5 groups*) Sex (k=1,2) Time (t=2013, 14, 15)	NSDUH MRB Statistical Inference Report [8]

Abbreviations:
- MA DPH: Massachusetts Department of Public Health data
- NSDUH: National Survey on Drug Use and Health
- MRB: Methodological Resource Book
- NB: Negative Binomial Distribution

* 5 age groups: 10 – 19, 20 – 24, 25 – 34, 35 – 49, 50 – 99
*** Other cause mortality is from Chapter 55 assuming that is no additional death in one year.

Table 2. Entering Cohort Counts by Year

Total counts (population size)	Year			
	**2012*****	**2013**	**2014**	**2015**
Population of OUD at time t ($\widehat{N}_{OUD,t}$)	166,835	226,861 (189832,301264)	233,184 (178671,271814)	275,070 (222160,357383)
Total number of non-FOD*deaths ($\widehat{N}_{D\_other,t}$)	596	964	1,332	1,605
Total number of FOD* deaths ($\widehat{N}_{FOD,t}$)	702	900	1,294	1,562
Entering Cohort ($N_{enter,t}$)	-	**10,943**	**8,187**	**44,512**

*** The estimates and observed data in year 2012 do not include data from Emergency Medical Services (EMS). Subsequent years do include those data.

© 2021 Linas BP et al. *JAMA Network Open.*

Natural History of OUD

RESPOND simulates opioid use as a series of transitions through four opioid use health states: 1) Non-Active and 2) Active non-injection use, as well as 3) Non-Active and 4) Active injection use (*Figure 1*). Throughout the simulation, there is a multi-directional movement between OUD states.

Transitions between drug use compartments impact four important outcomes: 1) risk of overdose, 2) risk of death from competing causes, 3) health care utilization (cost), and 4) quality of life.

The primary sources of data for substance use transitions are studies from the medical literature.

Structural Assumptions:

- OUD is a remitting and relapsing process over a lifetime. There is no health state of OUD cure or permanent recovery.
- Transitions between OUD health states are not time updated.

Methodological Notes:

Table *3* presents the key parameters related to OUD transitions for no treatment.

- All Confidence Intervals (CIs) are 95%, namely calculated at α=5% level of significance.
- CIs for proportions p_E, p_F, p_B, p_H, and p_D are calculated using the normal approximation to binomial proportions.
- CIs for rates R_A, R_C, and R_D are provided from the manuscript and calculated assuming Poisson distribution.
- Weekly rates and proportions, calculated from the respective overall estimates, are converted to weekly transition probabilities as indicated in .

© 2021 Linas BP et al. *JAMA Network Open.*

Table 3. No-Treatment: Opioid Use Disorder Transition Parameters

Parameter	Description	Value	Method	Source
**No Treatment**				
R_A	Rate of active non-injection to active injection	4.6 per 100 PY (3.0 , 6.6)		Neaigus, A., et al. [9]
P_A	Probability of active non-injection to active injection	0.000884 (0.000577 , 0.001268)	Calculated from R_A: P_A= 1-exp{R_A/52}	
R_C	Rate of non-active non-injection to active non-injection	16 per 100 PY (12.0 , 20.5)		
R_G	Rate of non-active injection to active injection			
P_C	Probability of non-active non-injection to active non-injection	0.00307 (0.00230 , 0.00393)	Calculated from Rate(R_C): P= 1-exp{R_C/52}	
P_G	Probability of non-active injection to active injection			
p_B	Proportion of active injection to active non-injection	0.34		Shah, N.G., et al. [10]
P_B	Probability of active injection to active non-injection	0.00067 (0.00054 , 0.0008)	Calculated from p_B: 1-exp(x) where x = ln(1-p_B)/(12*52)	
p_E	Proportion of non-active injection to active non-injection	0.13		
p_F	Proportion of non-active non-injection to active injection			
P_E	Probability of non-active injection to active non-injection	0.000223 (0.000115 , 0.00034)	Calculated from p_E: 1-exp(x) where x = ln(1-p_E)/(12*52)	
P_F	Probability of non-active non-injection to active injection			
p_D	Proportion of active non-injection to non-active non-injection	0.03 (0.0175 , 0.0425)		Nosyk, B., et al. [11]
p_H	Proportion of active injection to non-active injection			
P_D	Probability of active non-injection to non-active non-injection	0.00058 (0.00032 , 0.00085)	Calculated from p_D: 1-exp(x) where x = ln(1-p_D)/(52)	
P_H	Probability of active injection to non-active injection			
**Post-Treatment**				
P_A, P_B, P_D, P_E, P_F, P_H,	Same estimates with no-treatment.			
p^*_C	Proportion of non-active non-injection to active non-injection	0.65	CIs are calculated using the normal approximation to binomial proportions.	Bailey et al. [11]
p^*_G	Proportion of non-active injection to active injection			
P_C	Probability of non-active non-injection to active non-injection	0.2308	Calculated from p: 1-exp(x) where x = ln(1-p)/4	
P_G	Probability of non-active injection to active injection			

© 2021 Linas BP et al. *JAMA Network Open.*

- p^*_C and p^*_G indicate the percentage of people relapsed within a month of discharge (after inpatient detoxification).
- The denominators for calculating weekly probabilities depend on whether the respective available proportion or rate estimates are yearly or monthly.

Care Delivery

RESPOND models OUD while engaged with treatment using the same 4-state opioid use simulation that it uses to model OUD without treatment. The 4-state OUD simulation is embedded within all treatment episodes (blocks), such that individuals may both remain engaged with treatment, but also experience periods of drug use relapse. Each treatment type has its own bi-directional transition probabilities between active and non-active use. The net movement between active and non-active use while engaged with treatment favors movement to non-active use over time.

RESPOND simulates treatment using the following parameters:

1. Probability of movement onto treatment from no treatment
2. Treatment initiation effect – the probability of ceasing active opioid use immediately after initiating treatment
3. Bi-directional movements between active and non-active opioid use while engaged with treatment
4. Probability of loss to follow-up

The population that is lost to follow-up (disengages from care) must pass through a "post-treatment period" before rejoining the simulation of OUD. The post-treatment period is a four-week time, immediately following discontinuation of a treatment, during which the risk of relapse to drug use is high, as is the risk of overdose. Population that survives the post-treatment period transitions back to the simulation of OUD without treatment.

Movement From no Treatment to Treatment Episodes

Structural Assumptions:

- Only population in active opioid use states seeks OUD treatment. Population that is not currently using opioids does not seek treatment.

The main source of data to inform the probability of transition from no treatment to a treatment episode is the MA PHD.

Methodological Notes:

© 2021 Linas BP et al. *JAMA Network Open.*

The weekly transition probability from no-treatment to treatment is calculated as:

$$\widehat{\mathbf{P}}_{\mathbf{NoTrt} \rightarrow \mathbf{Trt\,.q}} = 1\text{- exp } \left\{ \frac{\widehat{\mathbf{N}}_{\mathbf{Obs,NoTrt} \rightarrow \mathbf{Trt\,.q}}}{\widehat{\mathbf{N}}_{\mathbf{Total,NoTrt} \rightarrow \mathbf{Trt\,.q}}} \cdot \frac{1}{4} \right\} \qquad (2)$$

where

$\widehat{\mathrm{N}}_{\mathrm{Obs,NoTrt} \rightarrow \mathrm{Trt\,.q}}$: the observed number of people with OUD who transitioned from no-treatment to treatment q in January 2013

$\widehat{\mathrm{N}}_{\mathrm{Total,NoTrt} \rightarrow \mathrm{Trt\,.q}}$: the total number of people with OUD "*at risk*" of transitioning from no-treatment to treatment q in January 2013

The weekly transition probability $\widehat{\mathrm{P}}_{\mathrm{NoTrt} \rightarrow \mathrm{Trt\,.q}}$ is estimated using data from the MA PHD repository[1], and is stratified by age (16 groups: 5-year age-groups from 10-85, and >85years old), sex, and treatment (q= Detox, Residential, Mmt, Ntx, and Bup) (**Table 4**).

Table 4. Transition Probabilities From no Treatment to Treatment

Age	Sex	Transition to Treatment				
		Detox	Residential	Methadone	Naltrexone	Buprenorphine
10-14	Male	0.0037 (0.0001, 0.0203)	0.0037 (0.0001, 0.0203)	0.0037 (0.0001, 0.0203)	0.0037 (0.0001, 0.0203)	0.0037 (0.0001, 0.0203)
10-14	Female	0.0064 (0.0002, 0.0351)	0.0064 (0.0002, 0.0351)	0.0064 (0.0002, 0.0351)	0.0064 (0.0002, 0.0351)	0.0064 (0.0002, 0.0351)
15-19	Male	0.0027 (0.0011, 0.0056)	0.0023 (0.0009, 0.0051)	0.0004 (0, 0.0022)	0.0016 (0.0004, 0.004)	0.0023 (0.0009, 0.0051)
15-19	Female	0.0027 (0.0009, 0.0063)	0.0022 (0.0006, 0.0055)	0.0005 (0, 0.003)	0.0016 (0.0003, 0.0047)	0.0027 (0.0009, 0.0063)
20-24	Male	0.0054 (0.0043, 0.0066)	0.001 (0.0006, 0.0016)	0.0009 (0.0005, 0.0015)	0.0012 (0.0007, 0.0018)	0.0051 (0.0041, 0.0063)
20-24	Female	0.005 (0.0038, 0.0066)	0.0009 (0.0004, 0.0016)	0.0016 (0.0009, 0.0026)	0.001 (0.0005, 0.0019)	0.0057 (0.0044, 0.0073)
25-29	Male	0.0056 (0.0047, 0.0067)	0.001 (0.0007, 0.0015)	0.0016 (0.0011, 0.0022)	0.0007 (0.0004, 0.0011)	0.0062 (0.0052, 0.0073)
25-29	Female	0.0042 (0.0032, 0.0053)	0.0009 (0.0005, 0.0015)	0.0028 (0.002, 0.0038)	0.0007 (0.0003, 0.0012)	0.0065 (0.0053, 0.0079)
30-34	Male	0.0053 (0.0043, 0.0063)	0.0009 (0.0005, 0.0014)	0.002 (0.0014, 0.0026)	0.0006 (0.0003, 0.001)	0.0068 (0.0058, 0.008)
30-34	Female	0.0035 (0.0025, 0.0046)	0.0007 (0.0003, 0.0013)	0.0027 (0.0019, 0.0038)	0.0006 (0.0003, 0.0012)	0.0071 (0.0057, 0.0087)
35-39	Male	0.0049 (0.0038, 0.0061)	0.0009 (0.0005, 0.0015)	0.0018 (0.0012, 0.0026)	0.0007 (0.0003, 0.0012)	0.0068 (0.0056, 0.0083)
35-39	Female	0.003 (0.002, 0.0044)	0.0006 (0.0002, 0.0013)	0.0021 (0.0012, 0.0033)	0.0008 (0.0003, 0.0017)	0.0066 (0.005, 0.0085)

[1] DPH Chapter 55 Data warehouse, datasets used include: APCD, BSAS, CASEMIX, DEATH, BIRTH, MATRIS, and PMPD

© 2021 Linas BP et al. *JAMA Network Open.*

40-44	Male	0.0046 (0.0036, 0.006)	0.0008 (0.0004, 0.0015)	0.0017 (0.0011, 0.0025)	0.0006 (0.0003, 0.0012)	0.0061 (0.0048, 0.0076)
40-44	Female	0.0027 (0.0017, 0.0041)	0.0005 (0.0001, 0.0013)	0.0021 (0.0012, 0.0033)	0.0005 (0.0001, 0.0013)	0.0057 (0.0042, 0.0076)
45-49	Male	0.0038 (0.0028, 0.005)	0.0007 (0.0003, 0.0013)	0.0017 (0.001, 0.0025)	0.0005 (0.0002, 0.0011)	0.0052 (0.0041, 0.0066)
45-49	Female	0.0021 (0.0013, 0.0034)	0.0005 (0.0001, 0.0012)	0.0015 (0.0008, 0.0026)	0.0005 (0.0001, 0.0012)	0.0051 (0.0037, 0.0068)
50-54	Male	0.0032 (0.0023, 0.0044)	0.0005 (0.0002, 0.0011)	0.0012 (0.0007, 0.002)	0.0005 (0.0002, 0.0011)	0.0051 (0.0039, 0.0065)
50-54	Female	0.0016 (0.0009, 0.0027)	0.0002 (0, 0.0008)	0.0013 (0.0006, 0.0023)	0.0005 (0.0001, 0.0012)	0.0048 (0.0034, 0.0064)
55-59	Male	0.0025 (0.0016, 0.0037)	0.0004 (0.0001, 0.0011)	0.0013 (0.0007, 0.0023)	0.0004 (0.0001, 0.0011)	0.0048 (0.0035, 0.0063)
55-59	Female	0.001 (0.0004, 0.0022)	0.0003 (0, 0.0012)	0.0013 (0.0006, 0.0026)	0.0007 (0.0002, 0.0017)	0.0044 (0.0029, 0.0065)
60-64	Male	0.0018 (0.0009, 0.0034)	0.0004 (0, 0.0013)	0.0018 (0.0009, 0.0034)	0.0004 (0, 0.0013)	0.0044 (0.0028, 0.0065)
60-64	Female	0.0012 (0.0003, 0.0031)	0.0003 (0, 0.0017)	0.0009 (0.0002, 0.0027)	0.0003 (0, 0.0017)	0.004 (0.0021, 0.0068)
65-69	Male	0.0014 (0.0003, 0.0041)	0.0005 (0, 0.0026)	0.0014 (0.0003, 0.0041)	0.0005 (0, 0.0026)	0.0037 (0.0016, 0.0073)
65-69	Female	0.0011 (0.0001, 0.0039)	0.0005 (0, 0.003)	0.0005 (0, 0.003)	0.0005 (0, 0.003)	0.0038 (0.0015, 0.0078)
70-74	Male	0.001 (0, 0.0058)	0.001 (0, 0.0058)	0.001 (0, 0.0058)	0.001 (0, 0.0058)	0.0031 (0.0006, 0.0091)
70-74	Female	0.0009 (0, 0.005)	0.0009 (0, 0.005)	0.0009 (0, 0.005)	0.0009 (0, 0.005)	0.0045 (0.0014, 0.0104)
75-79	Male	0.002 (0, 0.0108)	0.002 (0, 0.0108)	0.002 (0, 0.0108)	0.002 (0, 0.0108)	0.0039 (0.0005, 0.014)
75-79	Female	0.0013 (0, 0.0074)	0.0013 (0, 0.0074)	0.0013 (0, 0.0074)	0.0013 (0, 0.0074)	0.0053 (0.0015, 0.0136)
80-84	Male	0.0062 (0.0007, 0.0221)	0.0031 (0.0001, 0.017)	0.0031 (0.0001, 0.017)	0.0031 (0.0001, 0.017)	0.0062 (0.0007, 0.0221)
80-84	Female	0.0026 (0.0003, 0.0094)	0.0013 (0, 0.0072)	0.0013 (0, 0.0072)	0.0013 (0, 0.0072)	0.0065 (0.0021, 0.0151)
85-99	Male	0.0035 (0.0001, 0.0192)	0.0035 (0.0001, 0.0192)	0.0035 (0.0001, 0.0192)	0.0035 (0.0001, 0.0192)	0.0069 (0.0008, 0.0248)
85-99	Female	0.0011 (0, 0.0063)	0.0011 (0, 0.0063)	0.0011 (0, 0.0063)	0.0011 (0, 0.0063)	0.0057 (0.0018, 0.0132)

Block Initiation Effect

The main source of data for the treatment initiation effect and for substance use transitions while engaged with buprenorphine, naltrexone, or methadone is the NIDA CTN urine toxicology data. The CTN trials collected routine periodic urine toxicology from all participants. While the published clinical trials results censored participants at the first relapse to drug use (the primary outcome of that trial), the trials continued to collect data from patients who experienced a relapse, such that the database includes

© 2021 Linas BP et al. *JAMA Network Open.*

longitudinal urine toxicology from patients who relapsed to active use, as well as some who remitted back to non-active use over the course of the trial. We are able to analyze those data in an “as treated” manner, such that RESPOND is able to estimate realistic movements between active and non-active drug use states among people who are taking a medication. In addition, while engaged with treatment, the population faces a risk of disengaging from care (loss to follow-up).

Methodological Notes:

Upon entering treatment, a proportion of the population immediately transitions from active to non-active opioid use. This proportion $\hat{p}_{Init\_Act \rightarrow NonAct}$ is stratified by treatment episode as follows:

- buprenorphine (Bup): 0.74, based on the proportion of observed negative (non-active) urine samples at week 1
- naltrexone (Ntx): 0.90, based on the proportion of observed negative urine samples at week 5
- methadone (Mmt): 0.57, based on the proportion of observed negative urine samples at week 5
- post-treatment: 0.73, based on reported proportion of people relapsed in Bailey et al. [12]

We assume a binomial distribution and we use the Wald’s method to calculate 95% CIs for the proportion $\hat{p}_{Act \rightarrow NonAct}$ representing the block initiation effect.

Table 5. Block Initiation Effects Parameters: Weekly Transition Probabilities

Initial OUD state	Transition to			
	Treatment			Post-treatment*
	Bup	Ntx	Mmt	
Active non-injection	0.257(0.204 , 0.309)	0.103(0.058 , 0.148)	0.433(0.403 , 0.462)	1
Active injection	0.257(0.204 , 0.309)	0.103(0.058 , 0.148)	0.433(0.403 , 0.462)	1
Non-active non-injection	N/A**	N/A**	N/A**	0.73(0.662 , 0.798)
Non-active injection	N/A**	N/A**	N/A**	0.73(0.662 , 0.798)

* These estimates are the same for Bup, Ntx, Mmt, and detox.

** There is no block initiation effect for population that is not currently using opioids, because only population that is currently using opioids seeks care in the model.

Transitions Between Active and Non-Active Opioid Use While Engaged With Treatment

We estimated Weekly OUD transition probabilities $\hat{p}_{Trt\_Act \rightarrow NonAct}$ using Multi-State Models (MSMs)[13].

We fit separate models for each treatment: buprenorphine (Bup), naltrexone (Ntx), and methadone (Mmt), using data from the National Institute of Drug Abuse Clinical Trials Network (NIDA CTN) [4-6].

© 2021 Linas BP et al. *JAMA Network Open.*

Structural Assumptions:

- Population engaged with treatment may move between active and non-active opioid use, but the population engaged with treatment does not change the route of administration of their opioid use. In other words, population that entered treatment using non-injection opioids will not escalate to injection drug use while still engaged with treatment (Core Simulation within OUD treatment episodes (blocks) - *Figure 2*).

Transition probabilities between active and non-active states are the same for both injection and non-injection drug use. This structural assumption is confirmed by the MSM estimates for buprenorphine and methadone models, in which route was included as a model covariate, but was not a significant predictor of transition rates

Methodological Notes:

- Each MSM includes age and sex as covariates.
- Age is included as a continuous covariate in the MSM model, thus allowing estimation of the transition probabilities for age bins in which data are not available. We consider five 5 age groups: 10–19, 20–24, 25–34, 35–49, and 50–99 years old.
- OUD transition for Buprenorphine and Methadone: We keep all the weekly MSM estimates of OUD transition probabilities except week 1, which is considered as block initiation.
- OUD transition for Naltrexone: We delete the estimates for the first 4 weeks due to the inaccurate results from detoxification. Week 5 is also excluded from the analysis, as it is considered as block initiation.
- Transition probabilities from non-active to active use are defined as: $\hat{p}_{Trt\_NonAct \rightarrow Act} = 1 - \hat{p}_{Trt\_Act \rightarrow NonAct}$

© 2021 Linas BP et al. *JAMA Network Open.*

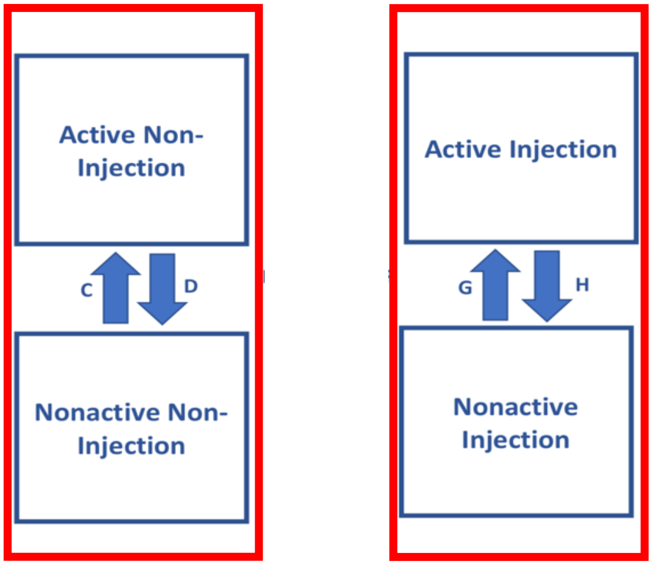

Figure 2. Core Simulation Within OUD Treatment Episodes (blocks)

Table 6. Transition Probabilities Between Active and Non-Active Opioid Use While Engaged with Treatment

Age	Sex	Initial OUD status	Treatment		
			Buprenorphine	Naltrexone	Methadone
10-19	Male	Active	0.750	0.847	0.669
10-19	Female	Active	0.746	0.711	0.692
10-19	Male	Nonactive	0.156	0.107	0.112
10-19	Female	Nonactive	0.118	0.130	0.089
20-24	Male	Active	0.738	0.832	0.678
20-24	Female	Active	0.735	0.687	0.700
20-24	Male	Nonactive	0.148	0.099	0.120
20-24	Female	Nonactive	0.112	0.119	0.095
25-39	Male	Active	0.723	0.814	0.688
25-39	Female	Active	0.721	0.656	0.710
25-39	Male	Nonactive	0.139	0.090	0.128
25-39	Female	Nonactive	0.105	0.108	0.102
40-54	Male	Active	0.686	0.763	0.713
40-54	Female	Active	0.683	0.573	0.732
40-54	Male	Nonactive	0.119	0.072	0.152
40-54	Female	Nonactive	0.090	0.083	0.121
55-99	Male	Active	0.628	0.673	0.746
55-99	Female	Active	0.626	0.443	0.762
55-99	Male	Nonactive	0.096	0.051	0.192
55-99	Female	Nonactive	0.072	0.056	0.154

Probability of Loss to Follow-Up

In the RESPOND model, every treatment episode has a corresponding post-treatment episode. The post-treatment block represents the period of time immediately following discontinuation of a medication or

© 2021 Linas BP et al. *JAMA Network Open.*

release from an abstinence-based setting (acute drug detoxification center, residential drug treatment, or jail), when opioid tolerance is low and the risk of overdose is higher than that of a person who never initiated treatment.

The main source of data for estimating the probability of loss to follow-up is Market Scan, a large insurance claims database containing millions of individuals who have commercial insurance coverage. As a randomized controlled trial, the CTN data cannot provide estimates of retention in care or loss to follow-up in the real world. We have previously published rates of loss to follow-up from buprenorphine and naltrexone treatment [14]. We therefore turn to Market Scan, which is nationally representative and reflects real-world practice in the U.S.

Structural Assumptions:

- In RESPOND, the only way to transition into a post-treatment episode is from a corresponding treatment episode.
- The "No Treatment" block does not have a post-treatment episode.
- RESPOND also considers the probability of immediate relapse to active opioid use upon being lost to follow-up from treatment:

Methodological Notes:

The weekly transition probability from treatment to post-treatment is calculated as:

$$\widehat{\mathbf{P}}_{\mathbf{Trt} \rightarrow \mathbf{Post-Trt}\,.\mathbf{q}} = 1 - \exp\left(\frac{r_q}{52}\right)$$

where

$r_q = \log\{1-(1-p_q)\}$ and p_q: the retention probability for treatment q.

Table *7* presents estimates of the weekly transition probabilities $\widehat{P}_{Trt \rightarrow Post-Trt\,.q}$ based on data from Morgan et al. [14], stratified by treatment.

Table 7. Weekly Transition Probabilities From Treatment q to Post-Treatment

**Treatment q**	$\widehat{\mathbf{P}}_{\mathbf{Trt} \rightarrow \mathbf{Post-Trt}\,.\mathbf{q}}$	**95% CI**	**Source**
Bup	0.0328	[0.0324, 0.0333]	Morgan et al. [14]
Naltrexone	0.0712	[0.0634, 0.0800]	
Methadone	0.0318	[0.0228, 0.0428]	

© 2021 Linas BP et al. *JAMA Network Open.*

Overdose

Every person who is actively using opioids faces the risk of overdose. The probability of overdose depends on age, sex, and route of drug use (injection vs. non-injection).

Structural Assumptions:

- Experiencing overdose has no independent impact on current or future opioid use behaviors.
- Only the population that is in an active opioid use state faces the risk of overdose.
- The risk of overdose is different between no-treatment, treatment, and post-treatment episodes.
- The risk of overdose is higher while engaged in treatment compared to not-engaged.

Methodological Notes:

Counts of overdose are a target for model calibration. **Table *8*** provides the empirically observed overdose fatalities from MA PHD.

Table 8. Opioid Overdoses

Year	Age	Sex	Total number of people with opioid overdose	Number of fatal opioid overdose	Number of people with non-fatal overdose	Total overdoses
2013	10-19	Male	88	7	86	89
2013	10-19	Female	77	1	76	77
2013	20-24	Male	542	50	507	547
2013	20-24	Female	346	18	337	350
2013	25-39	Male	1929	264	1746	1966
2013	25-39	Female	975	99	899	995
2013	40-54	Male	1147	240	966	1178
2013	40-54	Female	776	121	673	791
2013	55+	Male	705	70	647	713
2013	55+	Female	630	30	596	631
2014	10-19	Male	112	13	107	113
2014	10-19	Female	101	1	101	101
2014	20-24	Male	823	75	778	833
2014	20-24	Female	502	33	482	508
2014	25-39	Male	3174	421	2898	3268
2014	25-39	Female	1415	141	1313	1443

© 2021 Linas BP et al. *JAMA Network Open.*

2014	40-54	Male	1607	314	1368	1643
2014	40-54	Female	941	143	816	961
2014	55+	Male	913	105	825	928
2014	55+	Female	754	48	704	756
2015	10-19	Male	114	16	109	116
2015	10-19	Female	95	3	93	96
2015	20-24	Male	800	97	742	827
2015	20-24	Female	465	37	446	478
2015	25-39	Male	3804	545	3411	3914
2015	25-39	Female	1650	157	1537	1686
2015	40-54	Male	1863	357	1594	1919
2015	40-54	Female	973	142	871	1002
2015	55+	Male	1025	150	913	1045
2015	55+	Female	821	58	774	828

No Treatment

The rate $R_{OD,t}$ of overdose at time t for people not engaged in treatment is calculated as:

$$R_{OD,t} = \frac{N_{OD,t}}{N_{OUD,t} + \frac{1}{2} \cdot N_{enter,t}} \times 1\ \text{PY} \qquad (3)$$

for years t= 2013, 2014, 2015, assuming that each person contributes 1 person/year

where

$N_{OD,t}$: number overdose cases at time t

$N_{OUD,t}$: OUD cohort size at time t

$N_{enter,t}$: entering cohort size at time t

Overdose probabilities $P_{OD,t}$ are calculated from the respective overdose rates as:

$$P_{OD,t} = 1 - e^{-R_{OD,t}} \qquad (4)$$

Table *9* presents point estimates and 95% CIs of the overdose rates by age group, sex, OUD type, and year. We calculate 95% CIs for these rates assuming that the respective overdose counts $N_{OD,t}$ follow a Poisson distribution and using the normal approximation.

Table 9. Opioid Overdose Rates and 95% CIs by Age, Sex, Year, and Type of OUD

© 2021 Linas BP et al. *JAMA Network Open.*

Age	Sex	OUD	Years		
			2013	2014	2015
10-19	Male	Active_Noninjection	0.0004 (0.00027, 0.00057)	0.00018 (0.00012, 0.00024)	0.00046 (0.00031, 0.00061)
10-19	Male	Active_Injection	0.0025 (0.00188, 0.00316)	0.00108 (0.00084, 0.00133)	0.00278 (0.00216, 0.00341)
10-19	Female	Active_Noninjection	0.0005 (0.00028, 0.00063)	0.00058 (0.00038, 0.00077)	0.0014 (0.00091, 0.0019)
10-19	Female	Active_Injection	0.0027 (0.00199, 0.00348)	0.00348 (0.00265, 0.0043)	0.00845 (0.00638, 0.01053)
20-24	Male	Active_Noninjection	0.0008 (0.00071, 0.00096)	0.00108 (0.00095, 0.0012)	0.00092 (0.0008, 0.00103)
20-24	Male	Active_Injection	0.005 (0.00451, 0.00554)	0.00648 (0.00594, 0.00702)	0.00551 (0.00505, 0.00598)
20-24	Female	Active_Noninjection	0.0011 (0.00087, 0.00126)	0.00109 (0.00092, 0.00126)	0.00087 (0.00073, 0.00101)
20-24	Female	Active_Injection	0.0064 (0.00559, 0.00724)	0.00657 (0.00587, 0.00727)	0.00525 (0.00467, 0.00583)
25-39	Male	Active_Noninjection	0.0006 (0.00055, 0.00064)	0.00079 (0.00074, 0.00084)	0.00086 (0.00081, 0.00091)
25-39	Male	Active_Injection	0.0036 (0.0034, 0.00379)	0.00476 (0.00456, 0.00496)	0.00518 (0.00498, 0.00538)
25-39	Female	Active_Noninjection	0.0004 (0.00034, 0.00042)	0.00059 (0.00054, 0.00064)	0.00067 (0.00061, 0.00072)
25-39	Female	Active_Injection	0.0023 (0.0021, 0.00245)	0.00356 (0.00333, 0.00378)	0.00401 (0.00377, 0.00424)
40-54	Male	Active_Noninjection	0.0004 (0.00034, 0.00042)	0.00071 (0.00065, 0.00077)	0.00064 (0.00059, 0.0007)
40-54	Male	Active_Injection	0.0023 (0.00211, 0.00243)	0.00425 (0.00399, 0.0045)	0.00388 (0.00366, 0.00409)
40-54	Female	Active_Noninjection	0.0003 (0.00029, 0.00037)	0.0007 (0.00062, 0.00078)	0.00051 (0.00045, 0.00056)
40-54	Female	Active_Injection	0.002 (0.00181, 0.00215)	0.00422 (0.00389, 0.00455)	0.00306 (0.00282, 0.00329)
55-99	Male	Active_Noninjection	0.0003 (0.00025, 0.00032)	0.00045 (0.0004, 0.0005)	0.00049 (0.00044, 0.00055)
55-99	Male	Active_Injection	0.0017 (0.00155, 0.00186)	0.00272 (0.0025, 0.00293)	0.00298 (0.00276, 0.0032)
55-99	Female	Active_Noninjection	0.0003 (0.00025, 0.00032)	0.00015 (0.00013, 0.00017)	0.00016 (0.00014, 0.00018)
55-99	Female	Active_Injection	0.0017 (0.00155, 0.00188)	0.00091 (0.00083, 0.00099)	0.00099 (0.00091, 0.00107)

Overdose While on Treatment

The risk of overdose for people engaged in treatment, is derived by applying a multiplier parameter $m_{OD,q}$ to the respective no-treatment $R_{OD,t}$ estimates. i.e., the weekly overdose rate at time t for treatment q is:

$$R_{OD,t,q} = m_{OD,q} \times R_{OD,t} \qquad (5)$$

© 2021 Linas BP et al. *JAMA Network Open.*

where $R_{OD,t}$ is the no-treatment overdose rate at time t.

Table 10. Multipliers of Overdose Rates by Treatment q

Treatment q	$m_{OD,q}$	95% CI	Source
Buprenorphine	0.405	[0.35 , 0.46]	Morgan et al. [15]
Naltrexone	0.864	[0.42, 1.31] injectable	
Methadone	0.752	Non-parametric uncertainty distribution from bootstrapping of data	Morgan et al. [15] Sordo et al. [16]

Overdose During the Post-Treatment Period

During the post-treatment period, individuals faces a risk of overdose higher than that of people who never initiated a treatment.

The post-treatment rates of overdose at time t are calculated as:

$$R_{OD,\,t,\,post\text{-}trt} = \frac{R_{FOD,t}}{P_{FOD,t}} \times P_{act\_inj} \qquad (6)$$

where $R_{FOD,t}$ and $P_{FOD,t}$ are the post-detoxification fatal overdose rates and proportions respectively at time t (t=2013, 2014, 2015), P_{act_inj} is the proportion of active injectors, and 1-P_{act_inj} is the proportion of active non-injectors. We assume $P_{act\_inj} = 0.329$.

Table *11* presents the fatal overdose rates and probabilities by age group and sex.

Table 11. Fatal Overdose Parameters

**Rates within one month after discharge from detoxification**		
**Age**	**Male**	**Female**
**10-19**	0.0000	0.0000
**20-24**	0.0013	0.0029
**25-39**	0.0041	0.0020
**40-54**	0.0065	0.0050
**55+**	0.0031	0.0041
**Weekly Probabilities After Discharge from detoxification**		
	**Male**	**Female**
**10-19**	0	0
**20-24**	0.00032495	0.00072474
**25-39**	0.00102447	0.00049988

© 2021 Linas BP et al. *JAMA Network Open.*

**40-54**	0.00162368	0.00124922
**55+**	0.0007747	0.00102447

Mortality

RESPOND simulates mortality through two independent mechanisms, fatal opioid overdose and non-overdose death.

Fatal Overdose
The population that experiences overdose then faces a probability of death conditional on having had an overdose. This conditional probability of death, given an opioid overdose, is generalizable to all overdose cases and is therefore not stratified by age, sex, or OUD status. The population that survives an overdose does not change substance use as a result of the overdose.

The probability $P_{FOD,t}$ of fatal overdose at year t is calculated as:

$$P_{FOD,t} = \frac{N_{FOD,t}}{N_{OD,t}} \qquad (7)$$

where $N_{FOD,t}$ is the total number of fatal overdoses, and $N_{OD,t}$ is the total number of all-type overdoses.

**Table 12. Probabilities of fatal overdose.**	
**Year t**	$\mathbf{P_{FOD,t}}$
2013	0.1248 (0.1161 , 0.1338)
2014	0.1251 (0.1179 , 0.1324)
2015	0.1346 (0.1275 , 0.1417)

Non-overdose mortality
Non-overdose mortality reflects sex and type of OUD stratified competing risks of death among people who use drugs. Competing risks include mortality from conditions such as infectious endocarditis and sepsis, as well as medical comorbidities that accrue over a lifetime. The general approach to estimating competing risks of death is to apply standardized mortality ratios (SMRs) to actuarial lifetables for the U.S. reflecting elevated mortality among drug users.

© 2021 Linas BP et al. *JAMA Network Open.*

The SMR is calculated as:

$$SMR = \frac{N_{D\_other}}{R_D \times N_{OUD}} \qquad (8)$$

where N_{D_other} is the number of observed deaths not due to opioid overdose, R_D is the census death rate, and N_{OUD} is the size of the OUD population based on chapter 55 estimation.

We construct CIs around the SMRs estimates assuming that the N_{D_other} follows a Poisson distribution and using the normal approximation (Table 13).

Table 13. Standardized Mortality Rates (SMRs)

sex	OUD type	SMRs (95% CIs)
Male	Active - Non-injection	1.79(1.58 , 2.00)
Male	Active - Injection	4.41(3.84 , 4.98)
Male	Non-active – Non-injection	1.83(1.15 , 2.50)
Male	Non-active - Injection	4.59(2.71 , 6.46)
Female	Active – Non-injection	2.31(1.99 , 2.63)
Female	Active - Injection	5.67(4.81 , 6.53)
Female	Non-active – Non-injection	2.30(1.29 , 3.31)
Female	Non-active - Injection	5.62(2.87 , 8.37)

© 2021 Linas BP et al. *JAMA Network Open.*

eReferences

1. Siebert, U., et al., *State-Transition Modeling: A Report of the ISPOR-SMDM Modeling Good Research Practices Task Force-3.* Value in Health, 2012. **15**(6): p. 812-820.
2. Sonnenberg, F.A. and J.R. Beck, *Markov-Models in Medical Decision-Making - a Practical Guide.* Medical Decision Making, 1993. **13**(4): p. 322-338.
3. *Massachusetts Public Health Data Warehouse*. 2020 cited 2020 March 7]; Available from: https://www.mass.gov/public-health-data-warehouse-phd.
4. Lee, J.D., et al., *Comparative effectiveness of extended-release naltrexone versus buprenorphine-naloxone for opioid relapse prevention (X:BOT): a multicentre, open-label, randomised controlled trial.* Lancet, 2018. **391**(10118): p. 309-318.
5. Lee, J.D., et al., *NIDA Clinical Trials Network CTN-0051, Extended-Release Naltrexone vs. Buprenorphine for Opioid Treatment (X:BOT): Study design and rationale.* Contemp Clin Trials, 2016. **50**: p. 253-64.
6. Nunes, E.V., et al., *Ethical and clinical safety considerations in the design of an effectiveness trial: A comparison of buprenorphine versus naltrexone treatment for opioid dependence.* Contemporary clinical trials, 2016. **51**: p. 34-43.
7. Barocas, J.A., et al., *Estimated Prevalence of Opioid Use Disorder in Massachusetts, 2011-2015: A Capture-Recapture Analysis.* American Journal of Public Health, 2018. **108**(12): p. 1675-1681.
8. Substance Abuse and Mental Health Services Administration, R., MD, *2015 National Survey on Drug Use and Health: Methodological Resource Book* Center for Behavioral Health Statistics and Quality 2017(Section 13, Statistical Inference Report).
9. Neaigus, A., et al., *Transitions to Injecting Drug Use Among Noninjecting Heroin Users: Social Network Influence and Individual Susceptibility.* JAIDS Journal of Acquired Immune Deficiency Syndromes, 2006. **41**(4): p. 493-503.
10. Shah, N.G., et al., *Longitudinal predictors of injection cessation and subsequent relapse among a cohort of injection drug users in Baltimore, MD, 1988-2000.* Drug Alcohol Depend, 2006. **83**(2): p. 147-56.
11. Nosyk, B., et al., *Characterizing longitudinal health state transitions among heroin, cocaine, and methamphetamine users.* Drug Alcohol Depend, 2014. **140**: p. 69-77.
12. Bailey, G.L., D.S. Herman, and M.D. Stein, *Perceived relapse risk and desire for medication assisted treatment among persons seeking inpatient opiate detoxification.* J Subst Abuse Treat, 2013. **45**(3): p. 302-5.
13. Jackson, C., *Multi-State Models for Panel Data: The msm Package for R.* 2011, 2011. **38**(8): p. 28.
14. Morgan, J.R., et al., *Injectable naltrexone, oral naltrexone, and buprenorphine utilization and discontinuation among individuals treated for opioid use disorder in a United States commercially insured population.* J Subst Abuse Treat, 2018. **85**: p. 90-96.
15. Morgan, J.R., et al., *Overdose following initiation of naltrexone and buprenorphine medication treatment for opioid use disorder in a United States commercially insured cohort.* Drug and Alcohol Dependence, 2019. **200**: p. 34-39.
16. Sordo, L., et al., *Mortality risk during and after opioid substitution treatment: systematic review and meta-analysis of cohort studies.* BMJ, 2017. **357**: p. j1550.

© 2021 Linas BP et al. *JAMA Network Open.*
